# Supplementary material for: Architecture of the human G-protein-methylmalonyl-CoA mutase nanoassembly for B12 delivery and repair
Source: Nat Commun. 2023 Jul 19;14:4332. doi: 10.1038/s41467-023-40077-4 (PMC10356863; doi:10.1038/s41467-023-40077-4)
Supplement: Supplementary file 1 — Supplementary Information [file 41467_2023_40077_MOESM1_ESM.pdf]

Supporting Information

Architecture of the human G-protein-methylmalonyl-CoA mutase nanoassembly for B_{12} delivery and repair

Romila Mascarenhas[1#], Markus Ruetz[1#], Harsha Gouda[1], Natalie Heitman[1], Madeline Yaw[1] and Ruma Banerjee[1]

[1]Department of Biological Chemistry, University of Michigan, Ann Arbor, MI 48109

*Address correspondence to: Ruma Banerjee, E-mail: rbanerje@umich.edu

Supplementary Table 1. Crystallographic data for the human M_2C_2 complex

	MMUT•MMAA•GDP•CoA
Beamline	APS, GMCA-B
Wavelength (Å)	1.033
Temperature (K)	100
Space group	P 1 21 1
Cell dimension	
α, β, γ (º)	90, 105.5, 90
a, b, c (Å)	95.8, 221.8 121.8
Resolution (Å)	117.3-2.79 (3.07-2.79)
R_{merge} (%)	13.7 (77)
R_{meas} (%)	16.6(92)
R_{pim} (%)	9.2 (50)
<I/σ>	5.2 (1.6)
CC (½)	1.0 (0.73)
Completeness (%) (spherical)	61.9(9.1)
Completeness (%) (ellipsoidal)	89.3(63.9)
Multiplicity	3.1 (3.1)
No. Reflections	234358(8485)
No. Unique Reflections	7483 (2737)
Overall B (Å^2) (Wilson plot)	71.9
Resolution Range	80.6-2.79 (2.83-2.79)
Number of reflections (work/test)	74782/3731
R_{work} / R_{free} (%)	0.23/0.27
No. of atoms	
protein	30,235
water	14
Ligand: CoA	192
GDP	112
B-factors(Å^2)	
protein	63.82
water	48.57
Ligand: CoA	51.9
GDP	53.2
Rmsd deviations	
Bond lengths (Å)	0.004
Bond angles (º)	0.633
Ramachandran plot (%) Favored, allowed, outliers	96.04, 3.96, 0
MolProbity score	2.05
PDB code	8GJU

Supplementary Table 2: Summary of the biochemical penalties associated with MMUT and MMAA variants[1]

	GTPase activity (min^{-1})	Intrinsic GTPase activity (min^{-1})	Fold change	Activity (µmoles min^{-1} mg^{-1})	AdoCbl transferred	Cob(II)alamin transferred
wild type MMUT	2.1 ± 0.2 (n = 3)	N/A	36	89 ± 6 (n = 3)	blocked	complete
R228Q	0.7 ±0.1 (n = 4)	N/A	12	ND (n = 3)	partial	complete
R616C	0.4 ± 0.1 (n = 3)	N/A	7	71 ± 5 (n = 3)	complete	blocked
R694W	0.7 ± 0.3 (n = 3)	N/A	12	73 ± 1 (n = 3)	blocked	partial
R228Q/R616C	ND (n = 3)	N/A	ND	ND (n = 3)	complete	partial
R98G MMAA	0.08 ± 0.05 (n = 4)	0.05 ± 0.03 (n = 6)	2	N/A	complete	blocked
R209S MMAA	0.03 ± 0.01 (n = 4)	0.03 ± 0.02 (n=6)	1	N/A	complete	blocked

[1]The GTPase activity of MMAA in the presence of stoichiometric MMUT was measured. The fold change was calculated by dividing the GTPase activity in the presence of MMUT by the intrinsic GTPase activity of wild-type MMAA (0.06 ± 0.02 (n = 8)) or the MMAA variants in the absence of MMUT. n = number of repeats, ND = not detected, N/A = not applicable.

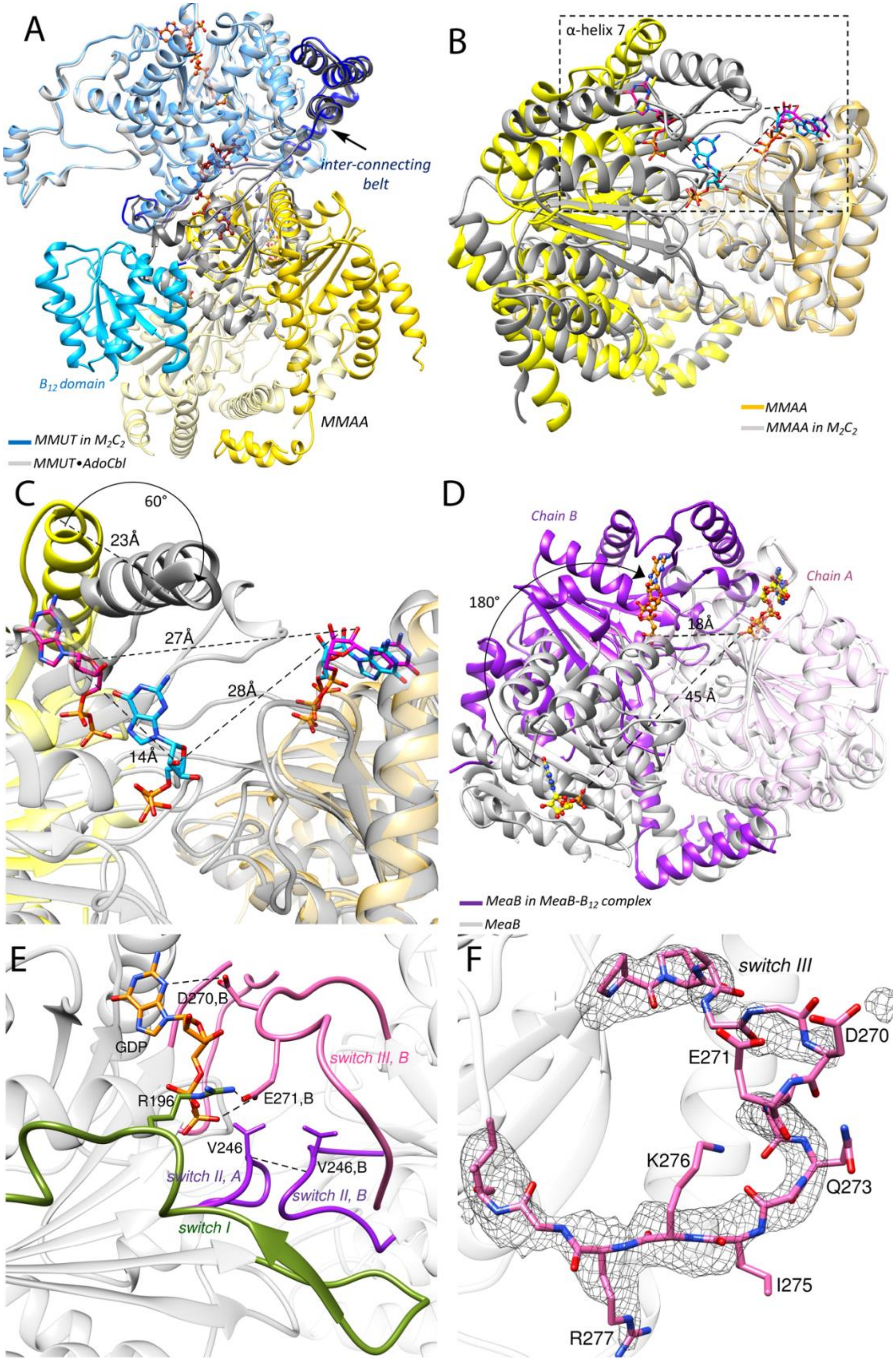
A
inter-connecting belt
B12 domain
MMAA
MMUT in M2C2
MMUT•AdoCbl
B
α-helix 7
MMAA
MMAA in M2C2
C
60°
23Å
27Å
28Å
14Å
D
Chain B
Chain A
180°
18Å
45 Å
MeaB in MeaB-B12 complex
MeaB
E
GDP
D270,B
switch III, B
R196
E271,B
V246
V246,B
switch II, A
switch II, B
switch I
F
switch III
D270
E271
K276
Q273
I275
R277

Supplementary Figure 1. A. Overlay of MMUT in M_2C_2 (blue ribbon) and MMUT·AdoCbl·malonyl-CoA (PDB: 2XIQ) shows the B_{12} domain becomes solvent exposed in M_2C_2 (cyan). MMAA (yellow ribbons) wedges between the B_{12} and substrate domains, forming new protein-protein interfaces. AdoCbl (red) and CoA (orange) are shown in stick display. **B.** Overlay of MMAA in the M_2C_2 complex (grey) with free MMAA (PDB: 2WWW) (yellow). **C.** A close-up of the boxed area in B shows that one subunit rotates by ~60° towards the dimer interface. GDP is shown in blue stick display in M_2C_2 and in magenta in free MMAA. **D**. Overlay of free MeaB (PDB:2QM7, grey ribbon) and MeaB in complex with the MutAB B_{12} domain (PDB:8DPB). The purple ribbon shows that chain B undergoes a 180° rotation upon complex formation. GDP in MeaB is shown as yellow sticks and GMPPCP in the MeaB-B_{12} domain is shown in orange. **E.** A close-up of the MMAA dimer interface shows that switch I (green), switch II (purple) and switch III from chain B (pink) are engaged in GDP (orange sticks) binding in the M_2C_2 complex. **F**. A 2mFo-DFc composite omit map at 1.5 σ of the switch III loop is shown in grey mesh. The backbone and side chains atoms of residues in the switch III loop are shown in pink stick display.

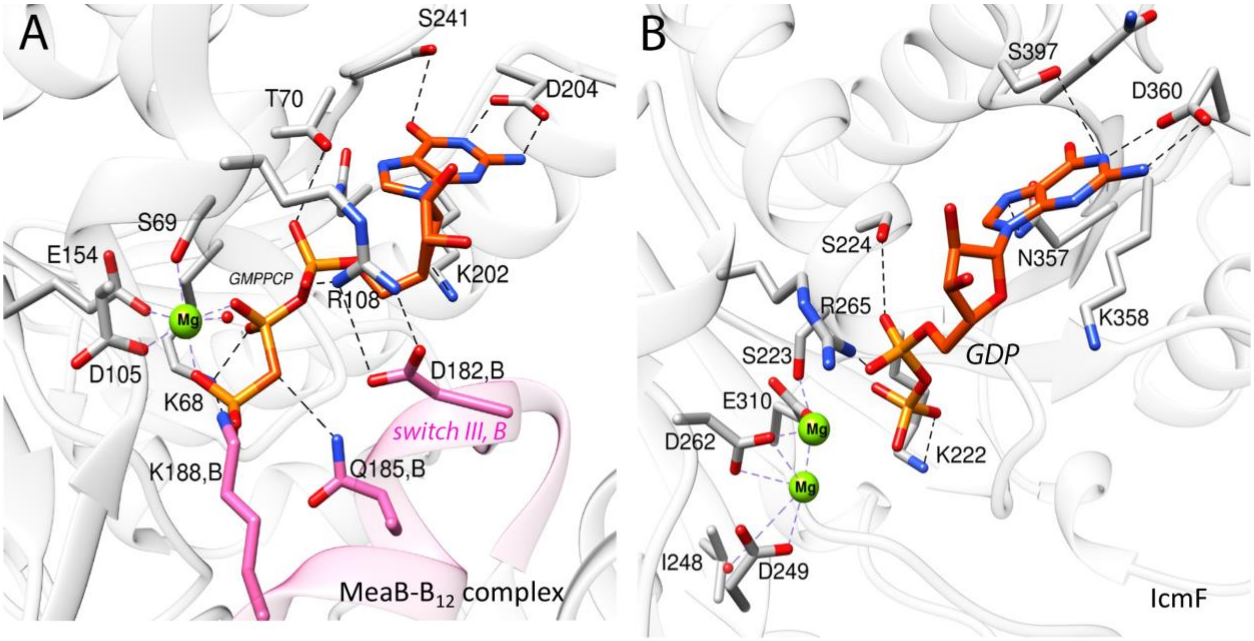

Supplementary Figure 2. **A**. Nucleotide binding site in the MeaB-MutAB B_{12} domain complex. Mg^{2+} (green sphere) is coordinated by oxygens from the α- and β-phosphate groups in GMPPCP (orange sticks), the oxygens from the side chains of Ser-69, Glu-154, Asp-105, and a water molecule. Switch III residues (pink) Gln-185 and Lys-188 from chain B form hydrogen bonds with GMPPCP, and Asp-182 forms a salt bridge with Arg-108. The corresponding residues in human switch III are Asp-270, Gln-273 and Lys-276. **B** The nucleotide binding site in IcmF. GDP is shown in orange sticks. Two Mg^{2+} ions (green spheres) are coordinated by Ser-223, Glu-310, Asp-262, and Asp-249.

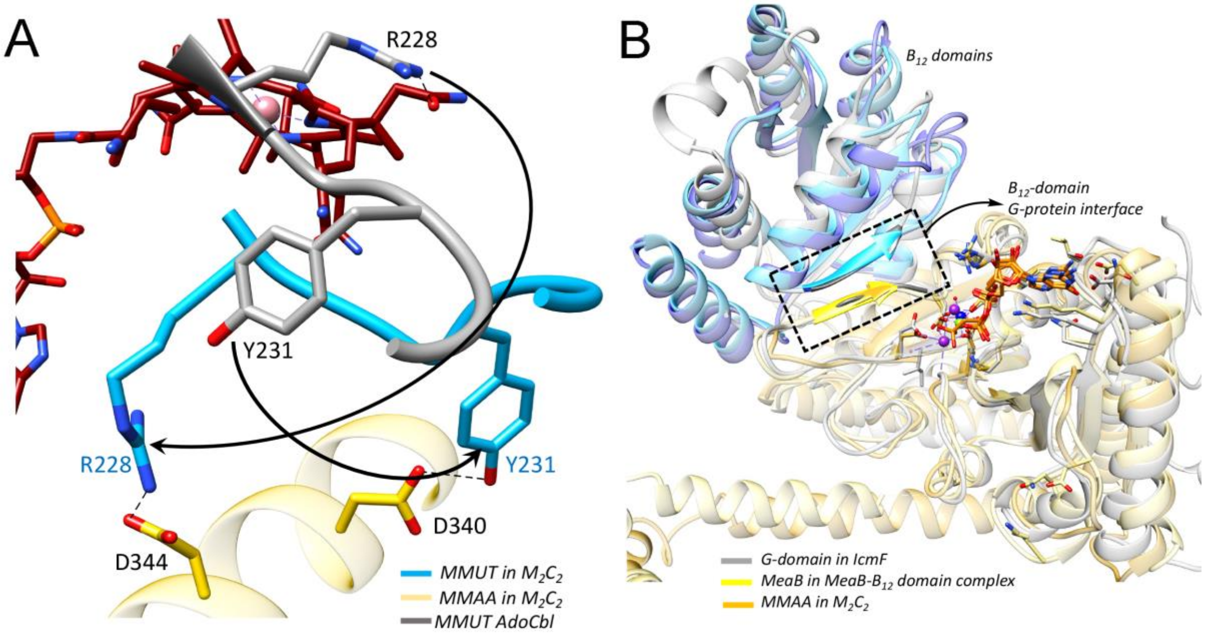

Supplementary Figure 3. A. Overlay of MMUT•AdoCbl (grey, PDB:2XIQ) and MMUT in the M_2C_2 complex (blue) showing reorientation of a loop (227-234), which positions Arg-228 and Tyr-231 for interactions with Asp-344 and Asp-340 in MMAA. Cobalamin is shown as red sticks. **B**. Overlay of the B_{12}- (blue) and G- (yellow) domains in M_2C_2 with the corresponding domain in IcmF (grey, PDB: 4XC8) and the MeaB-MutAB B_{12} domain complex (PDB:8DPB) reveals conservation in their juxtaposition. The boxed region highlights the β-sheet extension between switch I and the terminal β-sheet in the B_{12}-domain. GDP or GMPPCP is shown as orange sticks. Mg is shown as green, purple or dark blue spheres in MMAA, IcmF and MeaB, respectively.

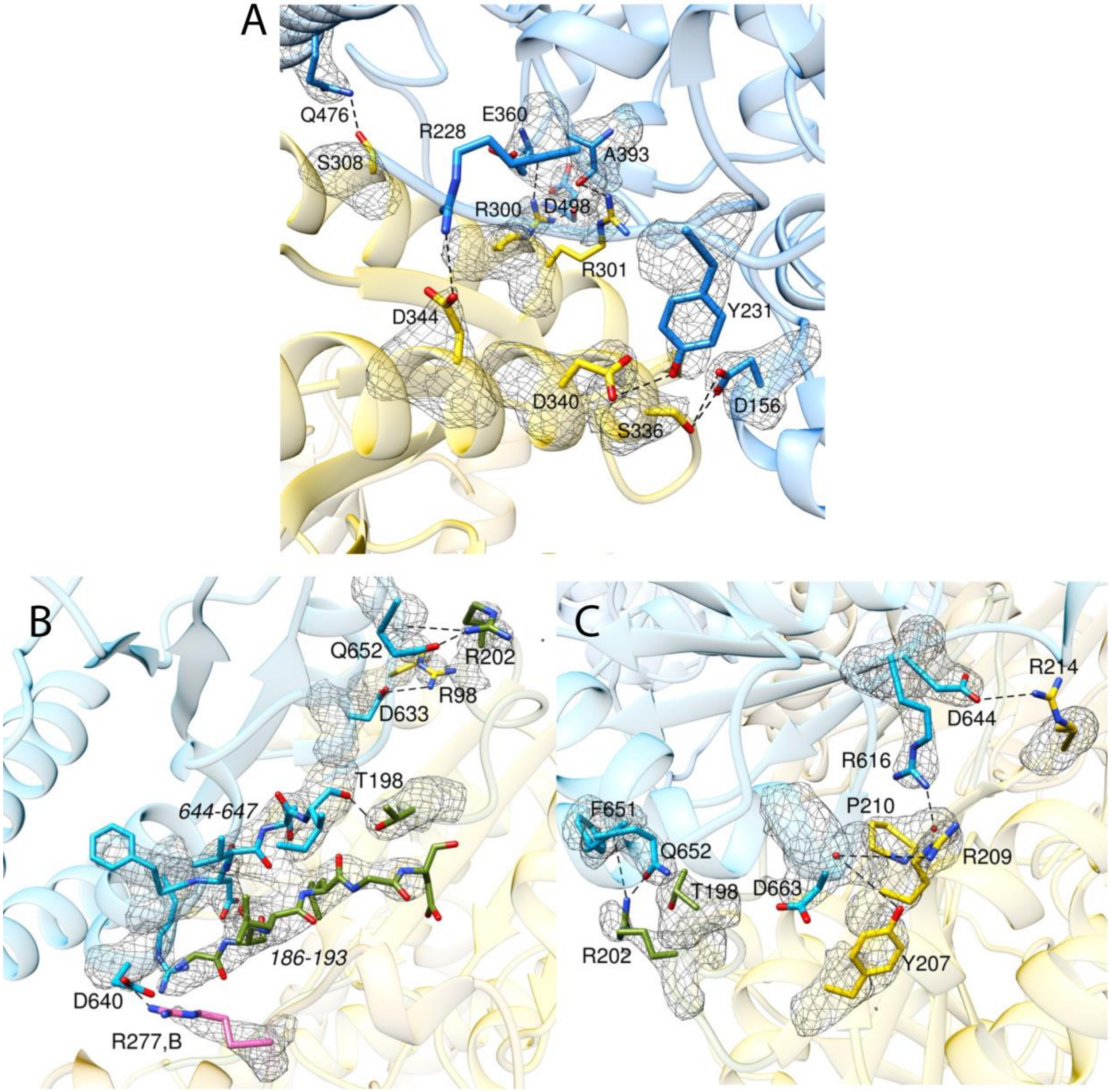

Supplementary Figure 4. A Residues interacting at the substrate domain (blue)-MMAA (yellow) interface are shown as sticks. 2mFo-DFc composite omit map of these residues is shown as a grey mesh at 1.5 σ. **B.C.** Residues interacting at the B_{12} domain (blue)-MMAA (yellow) interface and a 2mFo-DFc composite omit map of these residues is shown as a grey mesh at 1.5 σ. Residues on the B_{12}-domain and MMAA are shown as blue and yellow sticks, respectively, Switch I residues are shown as green sticks and Arg-277 from switch III is in pink.

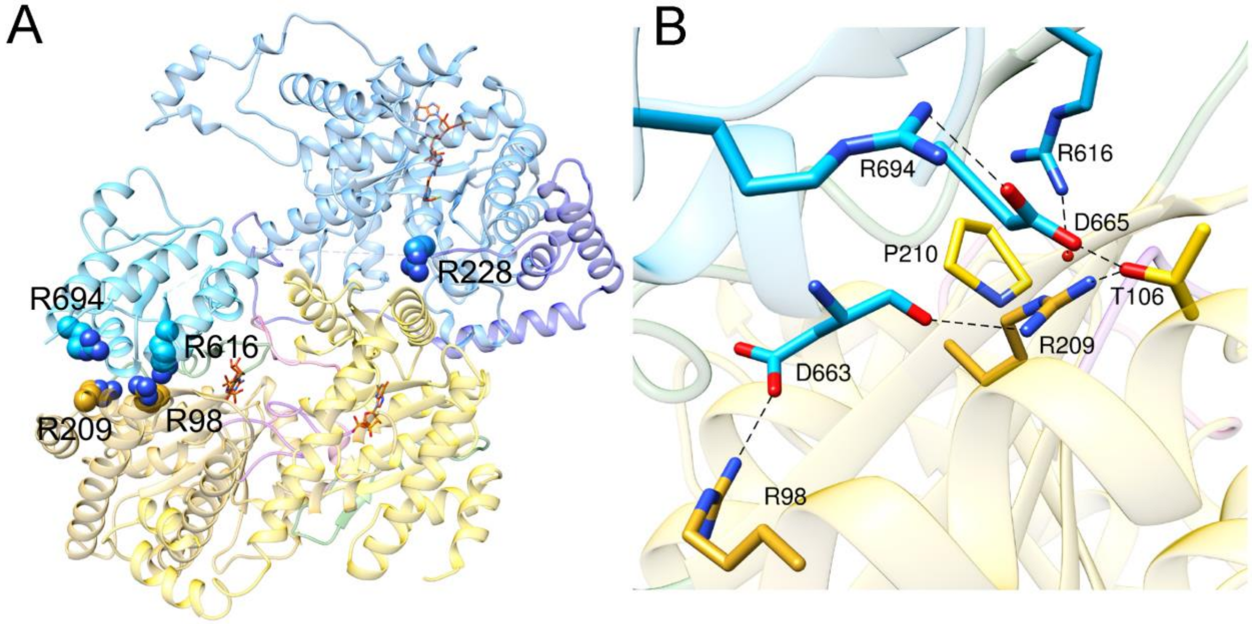

Supplementary Figure 5. Patient mutations at the MMUT•MMAA interfaces. **A.** Arg-228 resides at the substrate domain-MMAA interface while Arg-616 and Arg-694 in the B_{12}-domain are involved in a different interface with the second subunit of MMAA. Arg-98 and Arg-209 from MMAA also reside at the B_{12}-domain-MMAA interface. **B.** Close-up of the B_{12} domain-MMAA interface showing interactions between Arg-616 and the backbone of Pro-210. The sidechain of Asp-665 bridges Arg-694 to MMAA via a salt bridge with Arg-694 and a hydrogen bond to Thr-106. Arg-209 and Arg-98 form hydrogen bonds with the backbone and sidechain of Asp-663.

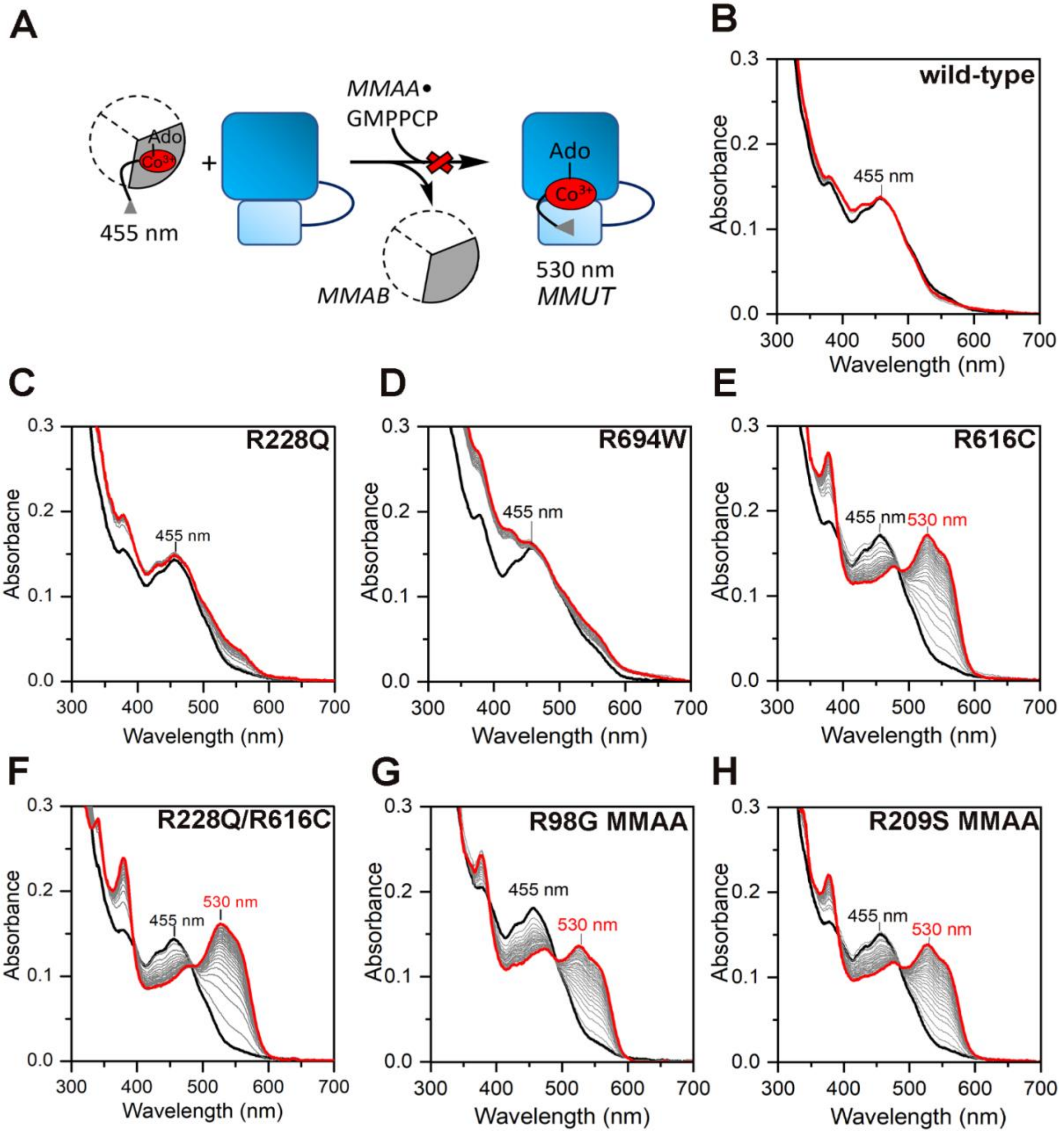

Supplementary Figure 6. AdoCbl transfer from MMAB to MMUT in the presence of MMAA•GMPPCP. (A) Scheme showing the gated transfer of AdoCbl from MMAB to MMUT, which does not occur when the non-hydrolysable GTP analogue GMPPCP is bound to MMAA. When mutations disrupt M_2C_2 complex formation, AdoCbl transfer from MMAB to MMUT is accompanied by a spectroscopic shift from 455 nm (MMAB•AdoCbl) to 530 nm (MMUT•AdoCbl). (B -H) Transfer of AdoCbl (15 µM) bound to MMAB (15 µM) in Buffer A following addition of wild-type or mutant MMUT (15 µM), wild-type or mutant MMAA (30 µM) and GMPPCP (1 mM). UV/visible spectra were recorded every minute for 30 min. The initial and final spectra are shown in black and red respectively. (**B**) wild-type MMUT, (**C**) R228Q MMUT, (**D**) R694W MMUT (**E**) R616C MMUT (**F**) R228Q/R616C MMUT, (**G**) R98G MMAA, and (**F**) R209S MMAA. The spectra are representative of three independent experiments.

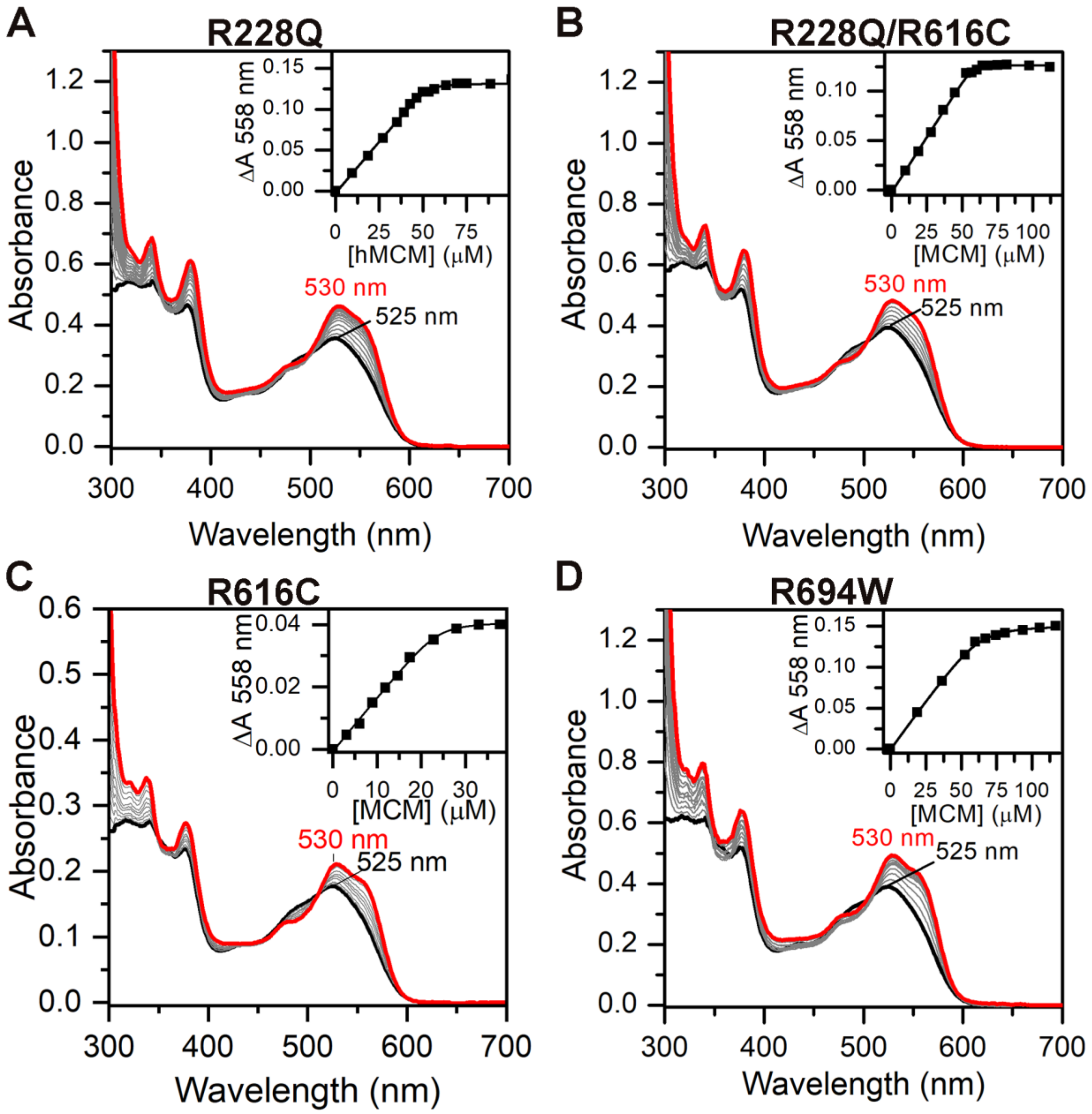

Supplementary Figure 7. Determination of dissociation constants for binding of free AdoCbl to MMUT. Varying concentrations of the MMUT variants were titrated into a solution of AdoCbl (50 µM) in Buffer A at 25 ˚C. (**A**) R228Q MMUT, (**B**) R228Q/R616C MMUT, (**C**) R616C MMUT, and (**D**) R694W MMUT. *Insets*. Dependence of A_{558nm} on MCM concentration. The data are representative of three independent experiments. Fitting of the binding isotherms in the insets to a single site binding model yielded the following $K_{D(AdoCbl)}$ values: wild type, R228W and R228W/R6161C =<0.2 µM, R6161C= 0.5 ± 0.2 µM, and R694W (1.1 ± 0.1 µM).

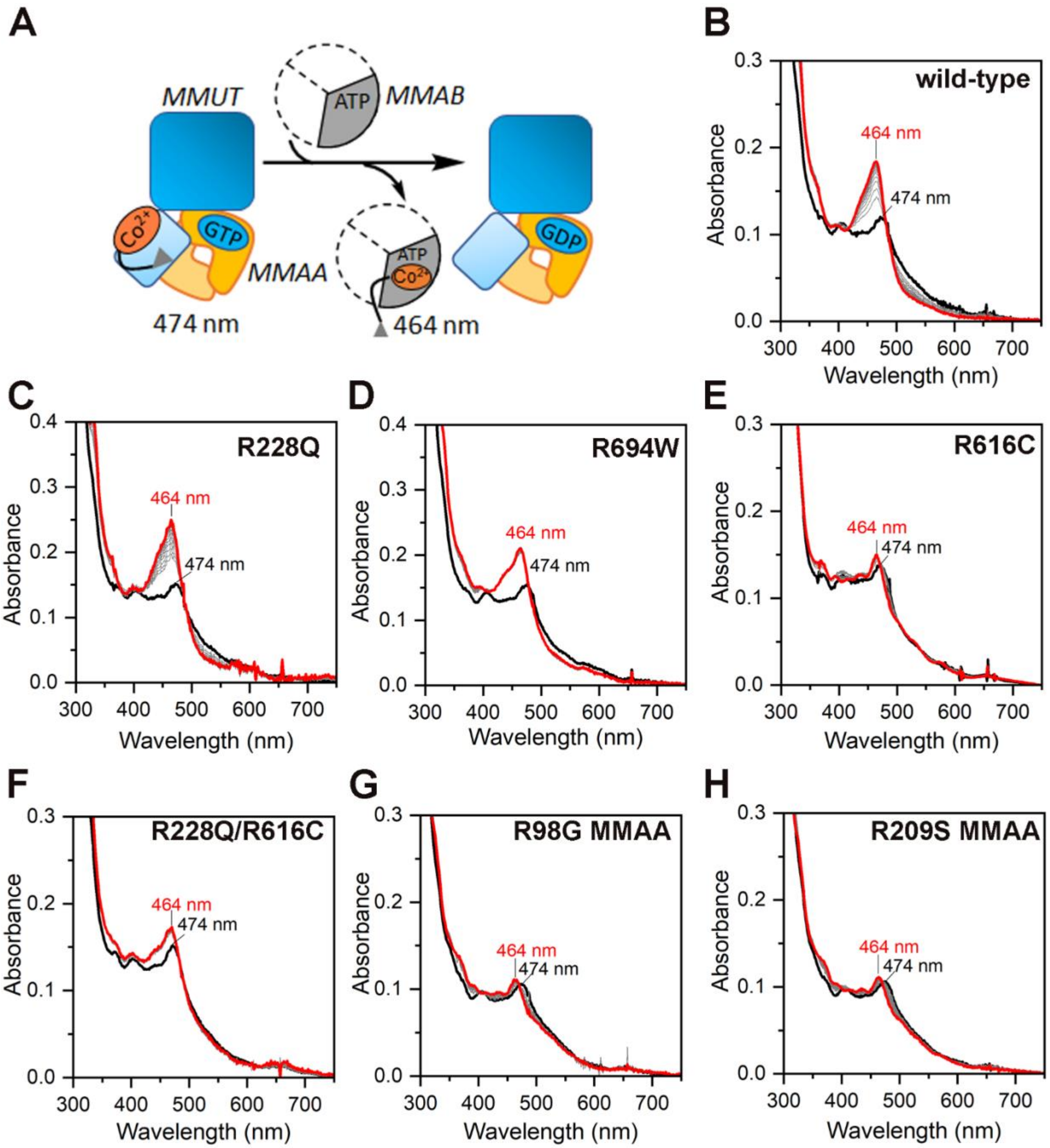

Supplementary Figure 8. Cob(II)alamin transfer from MMUT to MMAB. (**A**) Scheme showing the transfer of cob(II)alamin from MMUT to MMAB in the presence of MMAA•GTP. The blue shift from 474 nm to 464 nm with a concomitant increase in intensity is indicative of successful transfer. (**B-H**) Transfer of cob(II)alamin (15 µM) bound to wild-type or mutant MMUT (10 µM) in Buffer A following addition of MMAB (15 µM), wild-type or mutant MMAA (30 µM), 1 mM GTP and 5 mM ATP. UV/visible spectra were recorded every minute for 15 min; the initial and final spectra are shown in black and red, respectively. (**B**) Wild-type MMUT, (**C**) R228Q MMUT, (**D**) R694W MMUT, (**E**) R616C MMUT, (**F**) R228Q/R616C MMUT, (**G**) R98G MMAA, and (**H**) R209S MMAA. The spectra are representative of three independent experiments.

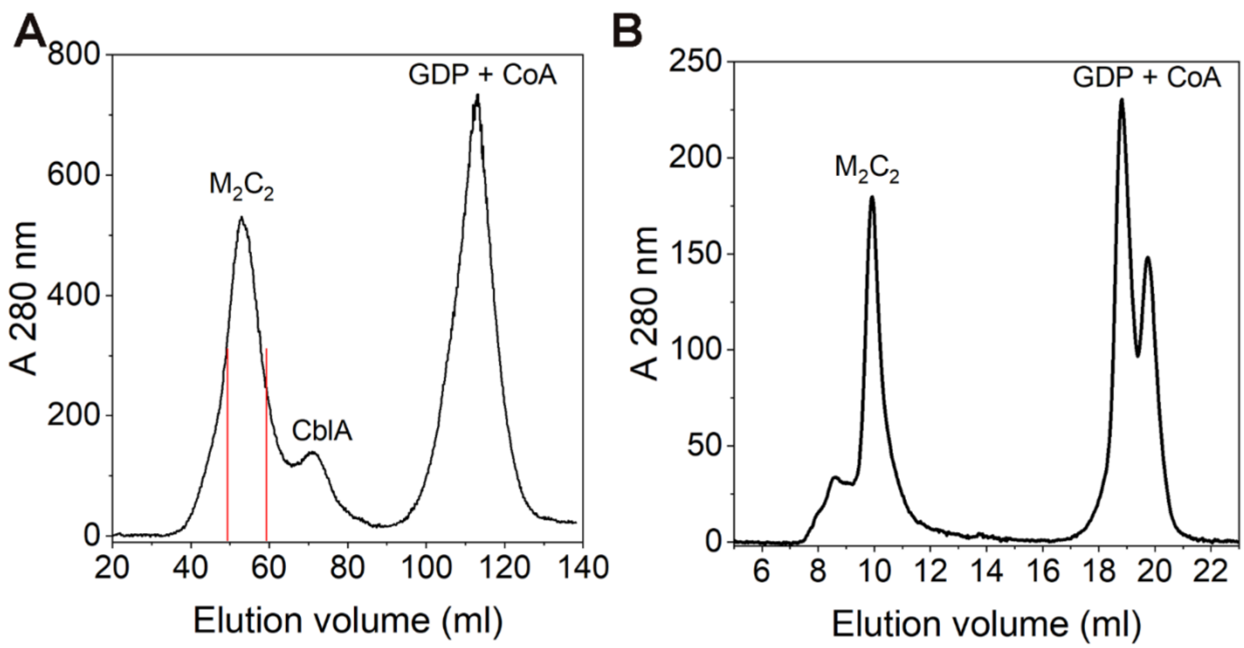

Supplementary Figure 9. Purification of the human M_2C_2 complex for crystallization. (**A**) Gel filtration profile of 100 µM MMUT, 200 µM MMAA, 2 mM GDP and 2 mM CoA on an S300 column. The M_2C_2 peak fractions (red bars) were used for crystallization. **(B).** The purified M_2C_2 complex from **A** was reanalyzed on an analytical S200 column, which demonstrated that the complex was intact.
