# Supplementary material for: Architecture of the human G-protein-methylmalonyl-CoA mutase nanoassembly for B12 delivery and repair
Source: Nat Commun. 2023 Jul 19;14:4332. doi: 10.1038/s41467-023-40077-4 (PMC10356863; doi:10.1038/s41467-023-40077-4)
Supplement: Supplementary file 3 — Description of Additional Supplementary Files [file 41467_2023_40077_MOESM3_ESM.docx]

**Supplementary Movie 1.** A morph video was generated using the structure of MMUT-AdoCbl (PDB:2XIJ) and MMUT in the M_2_C_2_ complex to show the large conformational change in the B_12_-domain upon complex formation. The swinging out of the B_12_-domain causes the two domains to become unstacked. The substrate-binding (light blue), B_12_-binding (dark blue) and interconnecting belt (green) are represented as ribbons.

**Supplementary Movie 2.** A morph video was generated using the structure of MMAA (PDB: 2WWW) and MMAA in the M_2_C_2_ complex to show the conformational changes in both chains upon complex formation. Chain A (dark shade) and chain B (light shade) are shown as yellow ribbons. The rotation of the two domains creates a complete GDP binding site at the dimer interface.
