# Supplementary material for: Architecture of the human G-protein-methylmalonyl-CoA mutase nanoassembly for B12 delivery and repair
Source: Nat Commun. 2023 Jul 19;14:4332. doi: 10.1038/s41467-023-40077-4 (PMC10356863; doi:10.1038/s41467-023-40077-4)
Supplement: Supplementary file 6 — Reporting Summary [file 41467_2023_40077_MOESM6_ESM.pdf]

nature portfolio

Corresponding author(s): Dr. Ruma Banerjee

Last updated by author(s): YYYY-MM-DD

Reporting Summary

Nature Portfolio wishes to improve the reproducibility of the work that we publish. This form provides structure for consistency and transparency in reporting. For further information on Nature Portfolio policies, see our Editorial Policies and the Editorial Policy Checklist.

Statistics

For all statistical analyses, confirm that the following items are present in the figure legend, table legend, main text, or Methods section.

n/a	Confirmed	
☐	☒	The exact sample size (*n*) for each experimental group/condition, given as a discrete number and unit of measurement
☐	☒	A statement on whether measurements were taken from distinct samples or whether the same sample was measured repeatedly
☒	☐	The statistical test(s) used AND whether they are one- or two-sided<br>*Only common tests should be described solely by name; describe more complex techniques in the Methods section.*
☒	☐	A description of all covariates tested
☒	☐	A description of any assumptions or corrections, such as tests of normality and adjustment for multiple comparisons
☐	☒	A full description of the statistical parameters including central tendency (e.g. means) or other basic estimates (e.g. regression coefficient) AND variation (e.g. standard deviation) or associated estimates of uncertainty (e.g. confidence intervals)
☒	☐	For null hypothesis testing, the test statistic (e.g. *F*, *t*, *r*) with confidence intervals, effect sizes, degrees of freedom and *P* value noted<br>*Give P values as exact values whenever suitable.*
☒	☐	For Bayesian analysis, information on the choice of priors and Markov chain Monte Carlo settings
☒	☐	For hierarchical and complex designs, identification of the appropriate level for tests and full reporting of outcomes
☒	☐	Estimates of effect sizes (e.g. Cohen's *d*, Pearson's *r*), indicating how they were calculated

Our web collection on statistics for biologists contains articles on many of the points above.

Software and code

Policy information about availability of computer code

Data collection	Agilent Chemstation B.03.01 (HPLC); LabSolutionsUVVis for Shimadzu UV-1900i or UV-2600 (UV-Vis assays); JBluIce Graphical User Interface (Crystallography)
Data analysis	OriginPro 2022 9.9.0.225 (UV-Vis assays); autoPROC 1. 0. 5 (20210420) and STARANISO Global Phasing Ltd. 2. 3. 73 (Crystallography); Phenix 1.15.2-3472 (Crystallography); CCP4 7.0 (Crystallography); Pymol 2.3.4 Schrodinger; UCSF Chimera 1.13.1, Coot 0.8.9.2; eLBOW in Phenix 1.15.2-3472 suit; Refmac in the CCP4 7.0 suit.

For manuscripts utilizing custom algorithms or software that are central to the research but not yet described in published literature, software must be made available to editors and reviewers. We strongly encourage code deposition in a community repository (e.g. GitHub). See the Nature Portfolio guidelines for submitting code & software for further information.

Data

Policy information about availability of data

All manuscripts must include a data availability statement. This statement should provide the following information, where applicable:

- Accession codes, unique identifiers, or web links for publicly available datasets
- A description of any restrictions on data availability
- For clinical datasets or third party data, please ensure that the statement adheres to our policy

All data are available in the manuscript or supplementary materials. The structure factors and coordinates for human MMUT•MMAA•CoA•GDP (PDB code: 8GJU) have been deposited in the Protein Data Bank. The following published structures were used in this study:

3BIC [https://doi.org/10.2210/pdb3BIC/pdb]
2XIJ [https://doi.org/10.2210/pdb2XIJ/pdb]
2XIQ [https://doi.org/10.2210/pdb2XIQ/pdb]
2WWW [https://doi.org/10.2210/pdb2WWW/pdb]
2QM7 [https://doi.org/10.2210/pdb2QM7/pdb]
8DPB [https://doi.org/10.2210/pdb8DPB/pdb]
4XC8 [https://doi.org/10.2210/pdb4XC8/pdb]

Research involving human participants, their data, or biological material

Policy information about studies with human participants or human data. See also policy information about sex, gender (identity/presentation), and sexual orientation and race, ethnicity and racism.

Reporting on sex and gender	'not applicable'
Reporting on race, ethnicity, or other socially relevant groupings	'not applicable'
Population characteristics	'not applicable'
Recruitment	'not applicable'
Ethics oversight	'not applicable'

Note that full information on the approval of the study protocol must also be provided in the manuscript.

Field-specific reporting

Please select the one below that is the best fit for your research. If you are not sure, read the appropriate sections before making your selection.

☒ Life sciences ☐ Behavioural & social sciences ☐ Ecological, evolutionary & environmental sciences

For a reference copy of the document with all sections, see nature.com/documents/nr-reporting-summary-flat.pdf

Life sciences study design

All studies must disclose on these points even when the disclosure is negative.

Sample size	No sample size calculation was performed
Data exclusions	No data were excluded from this study
Replication	Biochemical assays were performed with biological replicates of n=3 or greater or as stated in the manuscript. All replicates of the assay were successful.
Randomization	Not relevant to this study
Blinding	Blinding was not relevant to this study

Reporting for specific materials, systems and methods

We require information from authors about some types of materials, experimental systems and methods used in many studies. Here, indicate whether each material, system or method listed is relevant to your study. If you are not sure if a list item applies to your research, read the appropriate section before selecting a response.

Materials & experimental systems

n/a	Involved in the study	
☒	☐	Antibodies
☒	☐	Eukaryotic cell lines
☒	☐	Palaeontology and archaeology
☒	☐	Animals and other organisms
☒	☐	Clinical data
☒	☐	Dual use research of concern
☒	☐	Plants

Methods

n/a	Involved in the study	
☒	☐	ChIP-seq
☒	☐	Flow cytometry
☒	☐	MRI-based neuroimaging
